# Potential value of oral Mogibacterium in major depressive disorder

**DOI:** 10.3389/fcimb.2026.1816685

**Published:** 2026-04-24

**Authors:** Fengyun Wang, Guangjun Xi, En Zhao, Yongtao Yu, Lei Li, Mengmeng Zhong, Pan Wang, Yunuo Chen, Hui Weng, Lixiang Yang, Meilei Wang, Yachen Shi, Gaojia Zhang

**Affiliations:** 1 Department of Neurology, the Affiliated Wuxi People’s Hospital of Nanjing Medical University, Wuxi People’s Hospital, Wuxi Medical Center, Nanjing Medical University, Wuxi, China; 2 Comprehensive Stroke Center, the Affiliated Wuxi People’s Hospital of Nanjing Medical University, Wuxi People’s Hospital, Wuxi Medical Center, Nanjing Medical University, Wuxi, China; 3 Department of Gastroenterology, Xishan People’s Hospital of Wuxi City, Wuxi, China; 4 Department of Neurosurgery, the Affiliated Wuxi People’s Hospital of Nanjing Medical University, Wuxi People’s Hospital, Wuxi Medical Center, Nanjing Medical University, Wuxi, China; 5 Department of Psychology and Sleep Medicine, The Second Hospital of Anhui Medical University, Hefei, China; 6 Center for Scientific Research and Experiment, the Second Affiliated Hospital of Anhui Medical University, Hefei, China; 7 Department of Neurology, Xishan People’s Hospital of Wuxi City, Wuxi, China

**Keywords:** major depressive disorder, Mogibacterium, neuroplasticity, neurotrophin, oral microbiota

## Abstract

**Background:**

Major depressive disorder (MDD) is characterized by substantial clinical heterogeneity. Oral microbiota can provide real-time information relevant to the early identification of disease risk and the prediction of therapeutic outcomes. The present study aimed to characterize the specific profiles of oral microbiota in the buccal mucosa of patients with MDD and to explore potential mechanisms linking oral microbiota to the pathophysiology of MDD.

**Method:**

A total of 38 patients with MDD and 30 healthy controls (HCs) were enrolled. All MDD patients received standard antidepressant treatment and were followed up at two time points (2 weeks and 6 weeks). Neuropsychological assessments were administered, and 16S rRNA sequencing was employed to determine the abundance of oral bacteria.

**Results:**

(1) Significant differences in the diversity of oral microbiota from the buccal mucosa were observed between the MDD and HC groups. (2) The relative abundances of the oral genera *Aggregatibacter*, *Lautropia*, *Peptostreptococcus*, and *Mogibacterium* in the buccal mucosa were significantly altered in MDD patients compared to HCs. (3) The abundance of *Mogibacterium* was significantly correlated with scores on the 24-item Hamilton Depression Scale (HAMD-24) and the Self-Rating Depression Scale, as well as with serum levels of brain-derived neurotrophic factor (BDNF), nerve growth factor, and vascular endothelial growth factor (VEGF) in MDD patients. (4) BDNF and VEGF mediated the relationship between the relative abundance of oral *Mogibacterium* and HAMD-24 scores in MDD patients. (5) In MDD patients, the baseline relative abundance of oral *Mogibacterium* prior to treatment was significantly correlated with the rate of change in HAMD-24 scores following 2 and 6 weeks of antidepressant treatment.

**Conclusion:**

Oral *Mogibacterium* dysbiosis may contribute to the underlying pathophysiology of MDD, potentially via its influence on neuroplasticity. This oral bacterium may serve as a potential biomarker for diagnosing MDD and predicting responses to antidepressant treatment.

## Introduction

Major depressive disorder (MDD) is a prevalent mental health condition and a leading cause of disability worldwide (Marx et al., 2023). Its core clinical features include persistent low mood, diminished interest or pleasure, psychomotor slowing, anxiety, cognitive impairment, and insomnia-all of which substantially compromise personal wellbeing and quality of life (Jiao et al., 2025; Wang et al., 2024). Although the precise aetiology of MDD remains elusive, several pathophysiological mechanisms have been proposed, including monoaminergic dysfunction, hypothalamic-pituitary-adrenal axis dysregulation, neuroinflammation, impaired neuroplasticity, and genetic or epigenetic alterations (Malhi and Mann, 2018; Medina-Rodriguez et al., 2023; Réus et al., 2023). However, no single hypothesis adequately accounts for the full pathological complexity of MDD. It is increasingly recognised that MDD is unlikely to represent a unitary disorder; rather, it encompasses a heterogeneous spectrum of conditions spanning both adaptive physiological responses and maladaptive pathological states (Hu et al., 2024; Zhou et al., 2024). Given the considerable heterogeneity underlying the pathophysiology of MDD, the absence of effective biomarkers and the lack of individually tailored treatment strategies mean that patients often derive only limited benefit from current interventions (Perna et al., 2024; Sampogna et al., 2024; Xu et al., 2019).

The oral microbiome represents one of the most significant and complex microbial communities within the human body (Baker et al., 2024). Alterations in the oral microbiota associated with systemic disease states tend to be gradual yet reproducible (Peng et al., 2022; Santacroce et al., 2023). Since oral microbes can provide real-time insights into both health and disease, characterising the oral microbiome holds considerable promise for the early identification of disease risk and the prediction of therapeutic outcomes. Recent findings have indicated that oral microorganisms are involved not only in the pathogenesis of dental caries, periodontal diseases, and other oral conditions, but also in a variety of chronic diseases affecting the central nervous system (CNS) (Zhang et al., 2025). Meanwhile, the microbiota-oral-brain axis is increasingly regarded as a core underlying mechanism linking the oral microbiota to brain function (Lou et al., 2024). *Wang* et al. identified a significant association between the oral genera *Corynebacterium* and *Lautropia*, as well as the species *Lautropia mirabilis* and *Neisseria elongata*, and a reduced risk of ischaemic stroke in elderly Chinese women (Wang et al., 2023). Oral bacteria, including *Haemophilus*, *Neisseria*, *Actinobacillus*, and *Porphyromonas*, have been identified as dominant taxa associated with Alzheimer’s disease and mild cognitive impairment (Troci et al., 2024). Furthermore, the oral microbiome also appears to play an integral role in the aetiology and pathophysiology of various psychiatric disorders. The overall structure and composition of the oral microbiota in elderly patients with schizophrenia is characterised by a higher proportion of *Proteobacteria* and *Fusobacteria*, a lower proportion of *Bacteroidetes*, and a marked reduction in the relative abundance of anaerobic bacteria (Ling et al., 2023). In our previous study, an increased relative abundance of oral *Haemophilus* was associated with the severity of sleeplessness, suggesting that this oral microorganism may play a crucial role in the occurrence and progression of sleep disturbances in patients with MDD (Shi et al., 2025). Hence, the oral microbiome offers a novel avenue for identifying potential biomarkers to support the clinical practice of MDD.

Due to distinct niches within the oral cavity harbour characteristic microbial communities, these sites can be differentiated on the basis of their resident microbiota and broadly classified into three categories of microbial composition: dental plaque; the tongue and associated sites; and hard palate and buccal mucosa (Mark Welch et al., 2020). In previous studies, saliva samples obtained by spitting have been directly used to analyse the composition of oral microorganisms in patients with MDD (Lou et al., 2024; Simpson et al., 2020). However, salivary flow may affect the assessment of characteristic microbial communities residing at distinct oral sites. Additionally, in comparison with more commonly sampled sites, such as dental plaque, the throat, and the tongue dorsum, the hard palate and buccal mucosa have seldom been considered as sampling locations for investigating oral microbial communities (Zhang et al., 2025).

The present study aimed to identify the specific oral microbiota present in samples collected from the hard palate and buccal mucosa of patients with MDD. In addition, we sought to evaluate the associations between these microbiota and various clinical factors, including depression severity, levels of neurotrophin markers, and response to antidepressant therapy.

## Materials and methods

### Participants

A total of 38 MDD patients and 30 healthy controls (HCs) were enrolled from the Second Affiliated Hospital of Anhui Medical University. All MDD patients received a standardized antidepressant treatment, during which either a selective serotonin reuptake inhibitor (SSRI) or a serotonin-norepinephrine reuptake inhibitor (SNRI) was administered as monotherapy (please see **Table 1**). As the study’s primary aim was not to evaluate treatment efficacy, the design adopted an observational follow-up approach rather than a randomized controlled trial. All MDD patients were followed up at two time points after commencing medication: 2 weeks and 6 weeks.

**Table 1:** Clinical characteristics of all participants.

Variables	MDD (n = 38)	HCs (n = 30)	p-value
Age	26.58 ± 8.69	25.50 ± 3.78	0.319^a^
Sex (female/male)	29/9	19/11	0.243^b^
Education years	11.87 ± 2.72	13.47 ± 3.90	0.106^a^
Duration of the disease (month)	24.08 ± 27.98	–	–
BMI	22.49 ± 4.36	21.89 ± 4.23	0.569^c^
Before treatment			
HAMD-24 scores	27.16 ± 5.38	1.90 ± 1.45	< 0.001^a^
Anxiety	1.53 ± 1.48	0.90 ± 0.84	< 0.001^a^
Weight	3.29 ± 1.63	0.03 ± 0.18	< 0.001^a^
Cognitive disorder	1.21 ± 1.12	0.23 ± 0.43	< 0.001^a^
Retardation	3.16 ± 1.31	0.10 ± 0.31	< 0.001^a^
Circadian rhythms	3.95 ± 2.03	0.07 ± 0.25	< 0.001^a^
Somnipathy factor scores	4.66 ± 1.12	0.53 ± 0.78	< 0.001^a^
Hopelessness	9.37 ± 2.55	0.10 ± 0.31	< 0.001^a^
SDS scores	71.87 ± 10.43	38.23 ± 10.20	< 0.001^c^
PSQI scores	15.26 ± 2.08	4.23 ± 1.52	< 0.001^a^
Serum levels of BDNF	18.82 ± 6.02	28.57 ± 6.91	< 0.001^c^
Serum levels of IGF-1	176.25 ± 37.38	124.50 ± 16.70	< 0.001^c^
Serum levels of NGF	93.68 ± 24.66	127.02 ± 35.71	< 0.001^c^
Serum levels of VEGF	104.81 ± 26.43	70.70 ± 18.27	< 0.001^c^
Serum levels of S100β	88.32 ± 21.88	65.61 ± 27.57	0.001^c^
Serum levels of GFAP	0.21 ± 0.07	0.16 ± 0.03	< 0.001^c^
After treatment (2-week)			
HAMD-24 scores	9.68 ± 2.72	–	< 0.001^d^
SDS scores	57.82 ± 9.85	–	< 0.001^d^
PSQI scores	8.47 ± 2.85	–	< 0.001^d^
After treatment (6-week)			
HAMD-24 scores	7.24 ± 4.96	–	< 0.001^d^
SDS scores	54.84 ± 9.54	–	< 0.001^d^
PSQI scores	7.45 ± 2.68	–	< 0.001^d^
SSRIs or SNRIs, N%		–	–
Escitalopram	13 (34.21%)	–	–
Sertraline	9 (23.68%)	–	–
Duloxetine	8 (21.05%)	–	–
Fluoxetine	8 (21.05%)	–	–

MDD, major depressive disorder; HC, healthy control; BMI, body mass index; HAMD-24, 24-item Hamilton Depression Scale; SDS, Self-Rating Depression Scale; PSQI, Pittsburgh Sleep Quality Index; BDNF, brain-derived neurotrophic factor; IGF-1, insulin-like growth factor 1; NGF, nerve growth factor; VEGF, vascular endothelial growth factor; S100β, S100beta protein; SSRIs, selective serotonin reuptake inhibitors; SNRIs, serotonin-norepinephrine reuptake inhibitors.

a P-values were obtained by Mann-Whitney U test.

b P-values were obtained by Chi-square test.

c P-values were obtained by Independent-Samples T test.

d P-values were obtained by paired sample t test between before and after treatment in MDD patients.

The ethical approval was obtained from the Ethics Committee of the Second Hospital of Anhui Medical University (approval number: SL-YX2024-022). All participants or their legal guardians provided informed consent.

Patients with MDD were included according to the following criteria: 1) fulfillment of the Diagnostic and Statistical Manual of Mental Disorders, Fifth Edition (DSM-5) diagnostic criteria for MDD; 2) age between 19 and 50 years; 3) experiencing a first depressive episode; 4) either drug-naïve or having ceased all antidepressant medications for at least four weeks before study enrollment; and 5) no family history of psychotic disorders. Additionally, HCs were required to have no personal history of DSM-5 Axis I disorders, other psychiatric conditions, or major physical illnesses.

Exclusion criteria of all participants included: 1) organic lesions of the CNS or neurodegenerative diseases; 2) mental disorders secondary to severe physical illnesses; 3) a history of alcohol or substance abuse or dependence; 4) major systemic diseases, including endocrine disorders, autoimmune diseases, or impaired hepatic or renal function; 5) any malignancy; 6) cerebral trauma; or 7) pregnancy or lactation.

### Neuropsychological assessments

The 24-item Hamilton Depression Scale (HAMD-24) and Self-Rating Depression Scale (SDS) were used to evaluate the depressive symptoms. Meanwhile, the seven HAMD-24 factor scores, *i.e.*, retardation, cognitive disorder, hopelessness, anxiety, somnipathy, circadian rhythms and weight are also calculated. Furthermore, Pittsburgh Sleep Quality Index (PSQI) was performed to measures sleep quality over the previous month. Neuropsychological assessments were performed at baseline for healthy controls, and at baseline and both follow-up time points for patients with MDD.

### Collection of oral samples and 16S rRNA gene sequencing

In this study, all subjects were instructed to maintain their usual dietary habits and avoid consuming foods or liquids (such as hot pot, alcoholic beverages, or non-alcoholic soda) with strong irritating sensations starting 14 days prior to sampling (Barajas-Torres et al., 2022). Additionally, the sampling was scheduled for the early morning, before toothbrushing and eating. Oral biospecimens were collected noninvasively from the buccal mucosa using a sterile cotton swab, with consecutive sampling performed on the hard palate and buccal mucosa. Then, a sterile cotton swab was placed into a sterile centrifuge tube containing 1.5 mL of Tris-EDTA buffer solution (Solarbio) and subsequently stored at -80 °C. The oral sample collection process was conducted before neuropsychological assessment for each participant.

Total microbial genomic DNA was obtained using the Bacterial DNA Extraction Mini Kit (Mabio, Guangzhou, China) according to manufacturer’s instructions. 16S rRNA gene sequencing was described in the **Supplementary Materials** and our previous study (Shi et al., 2025).

### Serum samples collection and detection of serum neurotrophin levels

Subjects fasted overnight prior to peripheral venous blood collection using serum (clot-activating) vacutainer tubes. All samples were processed within 30 minutes post-collection by centrifugation (3500 rpm, 10 minutes, 4 °C). Following centrifugation, the supernatant serum was harvested and archived at -80 °C for future analysis. All participants provided blood samples after neuropsychological assessment.

The serum levels of brain-derived neurotrophic factor (BDNF), insulin-like growth factor 1 (IGF-1), nerve growth factor (NGF), vascular endothelial growth factor (VEGF), S100beta protein (S100β), and glial fibrillary acidic protein (GFAP) were assayed in triplicate, using the commercial Enzyme-linked Immunosorbent Assay kits (FineTest, Wuhan, China; Catalog Number: EH0043 for BDNF, EH0165 for IGF-1, EH0242 for NGF, EH0327 for VEGF, EH0543 for S100β, and EH0410 for GFAP) in accordance with the manufacturer’s protocols. Both the inter-assay and intra-assay coefficients of variation were less than 5%.

### Statistical analysis

The data were analyzed utilizing SPSS version 22.0 (SPSS, Inc., Chicago, IL, USA) and R sofware package (version 4.2.1). A two-tailed p-value < 0.05 was considered statistically significant.

#### Microbiota diversity analysis

Alpha diversity of the oral microbiota was evaluated using Observed species, Chao, and ACE indices to estimate community richness, and Shannon, Simpson, and Coverage indices to assess community diversity. Beta diversity analysis was performed using Partial Least Squares-Discriminant Analysis (PLS-DA) to examine compositional differences between groups (Shi et al., 2023b).

#### Statistical tests

Data normality was examined using the Kolmogorov-Smirnov test. Categorical variables were compared with the chi-square test. For continuous variables, group comparisons were made using the independent-samples t-test (normally distributed data) or the Mann-Whitney U test (non-normally distributed data). Pre- and post-treatment changes were assessed with the paired t-test. The diagnostic performance of oral microbiota for MDD identification was evaluated using Receiver Operating Characteristic (ROC) curves, with the area under the curve (AUC) calculated as a measure of accuracy. Optimal cutoff values were determined by maximizing the Youden index.

#### Correlation and mediation analysis

In MDD patients, partial correlation analysis was performed to examine associations between variables, adjusting for age, sex, education years, disease duration, and body mass index (BMI). A standard three-variable mediation model was employed to test whether oral microbiota mediated the relationship between neuropsychological assessment scores and serum molecular indicators, with the same set of covariates (Shi et al., 2023a, Shi et al., 2024). The rate of change for assessment scores was calculated as: (pre-treatment score - post-treatment score)/pre-treatment score (He et al., 2024, He et al., 2025).

## Results

### Clinical features of participants

As shown in **Table 1**, the two groups did not differ significantly in age, sex, years of education, or BMI. Compared with HCs, MDD patients had significantly higher total scores on the HAMD-24, SDS, and PSQI, as well as higher scores across all seven factors of the HAMD-24 (**Table 1**). Additionally, significant between-group differences were observed in serum levels of BDNF, IGF-1, NGF, VEGF, S100β and GFAP (**Table 1**).

### Compositional analysis of oral microbiota and diversity analysis

A total 3,780,462 sequences (SRA accession number: PRJNA1436510) were obtained from 68 samples using QIIME sofware (version 1.91; URL link: https://qiime.org/). were assigned based on a threshold of 97% sequence similarity. The MDD group demonstrated a greater abundance of OTUs compared to the HC group, with a count of 1016 versus 1020, respectively, encompassing 706 shared OTUs (**Supplementary Figure 1**). The rarefaction curves for the samples reached a saturation plateau at a sequencing depth of 37,764 reads, indicating that the majority of microbial species were adequately covered by the sequencing depths employed, and that the sample size was appropriately sized (**Supplementary Figure 2**).

**Figure 1:**
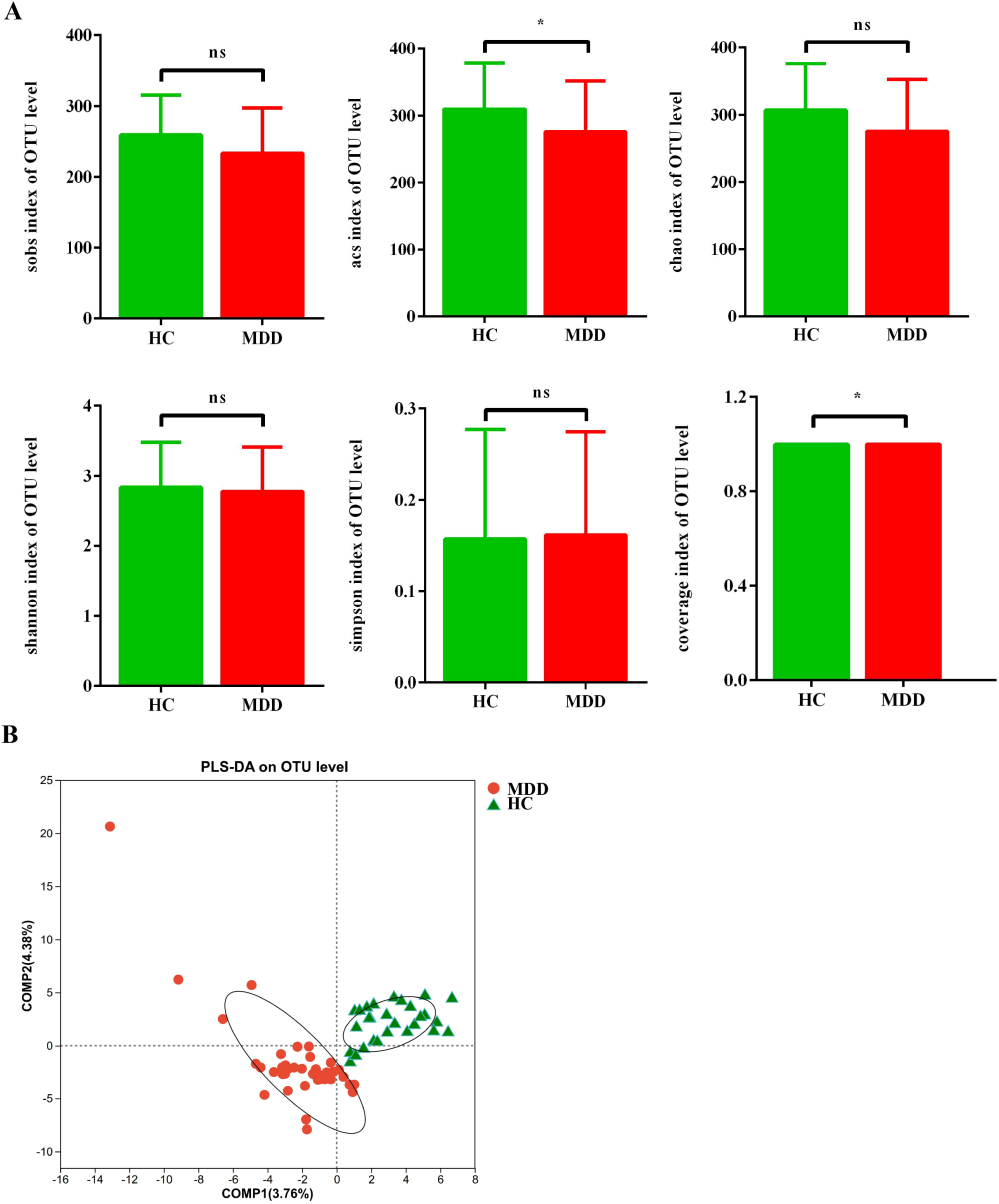
Alpha-diversity and Beta-diversity indices for the species in the oral microbiota between MDD and HC groups. **(A)** α-diversity indices. ^*^p < 0.05, ^**^p ≤ 0.01, ^***^p ≤ 0.001. **(B)** β-diversity indices. The scale stands for the relative distance without practical significance. MDD, major depressive disorder; HC, healthy control; PLS-DA, Partial Least Squares-Discriminant Analysis.

Analysis of alpha diversity using six indices (Sob, Chao, ACE, Shannon, Simpson, and Coverage) revealed that only ACE and Coverage differed significantly between the MDD and HC groups (**Figure 1A**). Subsequently, beta diversity was evaluated by PLS-DA to account for inter-individual variability within groups (**Figure 1B**).

### Compositional analysis of oral microbiota at genus level levels between two groups

**Supplementary Figure 3** presents the 50 most prevalent bacterial genera, characterized by their highest relative abundances in both groups. These genera belong to nine distinct phyla: *Proteobacteria, Firmicutes*, *Bacteroidota*, *Fusobacteriota*, *Actinobacteriota*, *Cyanobacteria*, *Patescibacteria, Campilobacterota, and Spirochaetota*.

**Figure 2:**
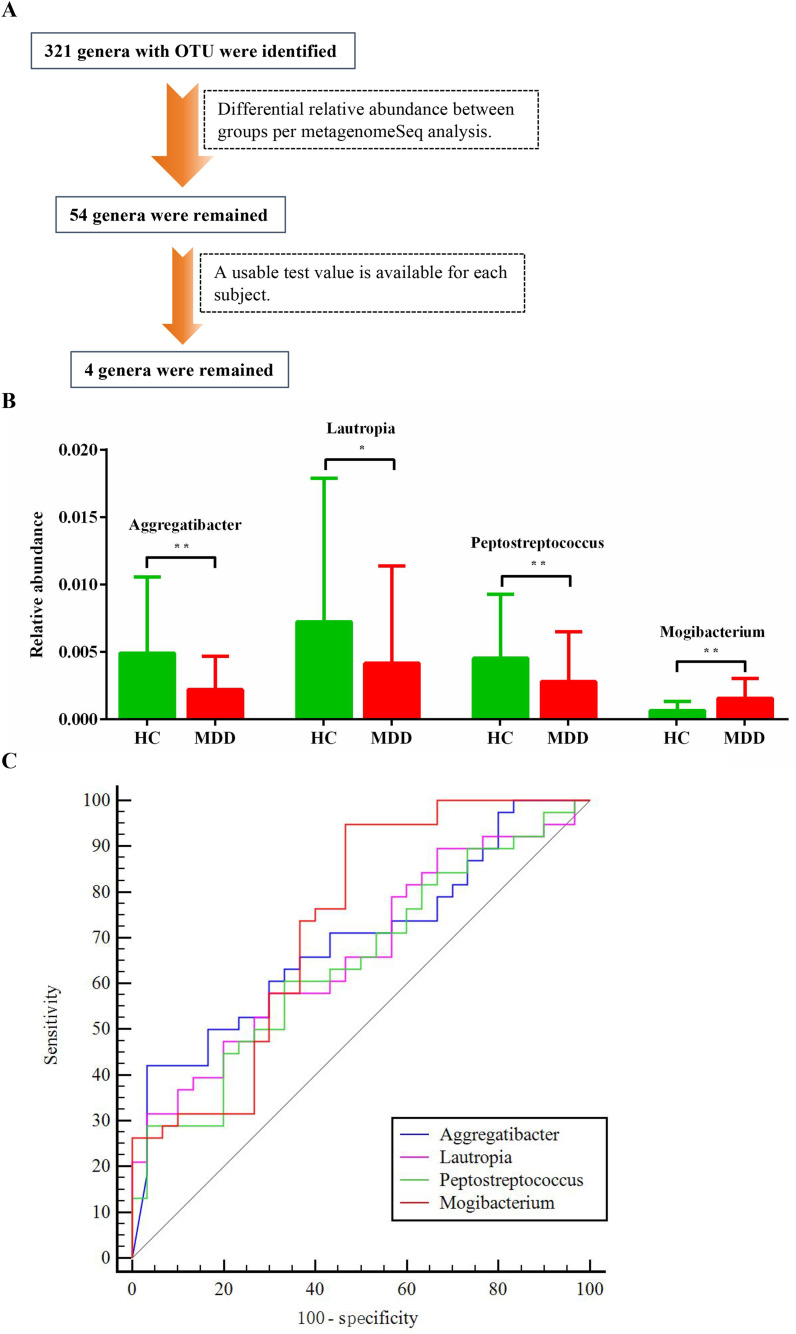
Differentiation of oral microbiota at the genus level in comparative expression analysis between MDD and HC groups. **(A)** Oral microbial screening process. **(B)** Taxa with significant differences at genus level between the MDD and HC groups. **(C)** ROC curve analysis. ^*^0.05 < p ≤ 0.01; ^**^0.01 p ≤ 0.001. MDD, major depressive disorder; HC, healthy control; ROC, receiver operating characteristic; AUC, area under the curve.

A total of 321 genera were identified in both the MDD and HC groups. Following MetagenomeSeq analysis, 54 genera exhibited statistically significant differences in relative abundance between the two groups (**Figure 2A**). To ensure analytical precision in evaluating group differences, four oral microbiota genera were retained for further analysis, as reliable measurements could be obtained consistently across all subjects (**Figure 2A**). Notably, these four oral genera were among the top 50 most prevalent bacterial genera (**Supplementary Figure 3**). Compared with the HC group, MDD patients showed a significant reduction in the relative abundance of *Aggregatibacter*, *Lautropia*, and *Peptostreptococcus*, while *Mogibacterium* was significantly increased (**Figure 2B**).

To evaluate the diagnostic potential of these four oral microbiota for MDD, ROC curve analysis was performed. *Mogibacterium* showed the highest discriminative ability, with an AUC of 0.732, a sensitivity of 94.74%, and a specificity of 53.33% (**Figure 2C**). The remaining three microbiota also demonstrated moderate diagnostic value, each achieving an AUC above 0.6 (**Figure 2C**, **Supplementary Table 1**).

### Association analysis of oral microbiota with clinical characteristics in MDD patients

The relative abundance of *Mogibacterium* showed significant positive correlations with HAMD-24, SDS, and PSQI scores, and was also associated with circadian rhythm disturbances factor scores, somnipathy factor scores, and hopelessness factor scores on the HAMD-24 scale (**Table 2**). In addition, the relative abundance of *Mogibacterium* was significantly negatively correlated with serum levels of BDNF and NGF, and significantly positively correlated with serum VEGF levels (**Table 2**). Meanwhile, a significant correlation was observed between the relative abundance of *Mogibacterium* and serum GFAP levels (r = 0.362, p = 0.026). These correlation analyses were adjusted for age, sex, years of education, disease duration, and BMI. However, no significant correlations were observed between these clinical characteristics and the relative abundance of *Aggregatibacter*, *Lautropia*, or *Peptostreptococcus*. To assess whether shared depressive symptom severity drives the co-variation between microbial and neurobiological variables, further partial correlation analyses were performed between *Mogibacterium* and neurotrophins, with HAMD-24 scores included as additional covariates. The results indicated no significant correlations between the relative abundance of *Mogibacterium* and serum levels of neurotrophins, except for NGF (**Supplementary Table 2**).

**Table 2:** Correlation analyses between relative abundance of Mogibacterium and clinical characteristics and serum neurotrophin levels in MDD patients.

Variables	r-value	p-value
Baseline		
HAMD-24 scores	**0.450**	**0.005**
Anxiety	0.014	0.933
Weight	0.223	0.177
Cognitive disorder	0.077	0.647
Retardation	-0.043	0.796
Circadian rhythms	**0.321**	**0.049**
Somnipathy factor scores	**0.360**	**0.026**
Hopelessness	**0.375**	**0.020**
SDS scores	**0.377**	**0.020**
PSQI scores	**0.451**	**0.004**
Serum levels of BDNF	**-0.413**	**0.010**
Serum levels of IGF-1	0.318	0.052
Serum levels of NGF	**-0.362**	**0.025**
Serum levels of VEGF	**0.377**	**0.020**
Serum levels of S100β	0.221	0.183
After treatment (2-week)		
Changed rate of HAMD-24 scores	**-0.419**	**0.009**
Changed rate of SDS scores	**-0.386**	**0.017**
Changed rate of PSQI scores	-0.092	0.584
After treatment (6-week)		
Changed rate of HAMD-24 scores	**-0.432**	**0.007**
Changed rate of SDS scores	**-0.373**	**0.021**
Changed rate of PSQI scores	-0.077	0.645

Age, sex, education years, body mass index, and duration of the disease were used as covariates. The bolded values denote statistically significant results.

MDD, major depressive disorder; HAMD-24, 24-item Hamilton Depression Scale; SDS, Self-Rating Depression Scale; PSQI, Pittsburgh Sleep Quality Index; BDNF, brain-derived neurotrophic factor; IGF-1, insulin-like growth factor 1; NGF, nerve growth factor; VEGF, vascular endothelial growth factor; S100β, S100beta protein.

**Figure 3:**
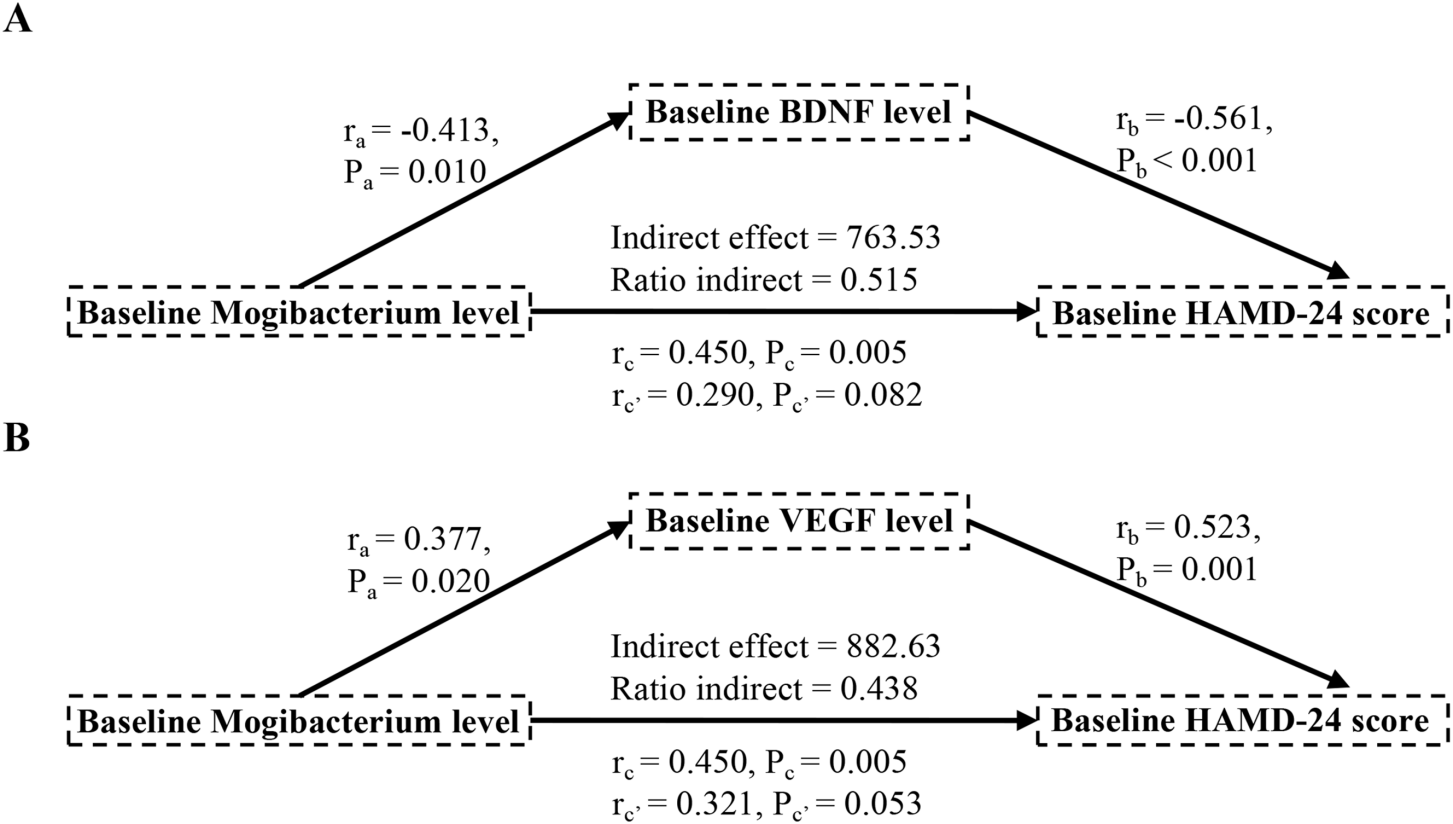
Associations of neuropsychological assessments and serum levels of indicators with oral *Mogibacterium* in MDD patients. Mediation analysis of the association between the relative abundance of oral *Mogibacterium* and depression assessments, examining the mediating role of serum BDNF and VEGF. MDD, major depressive disorder; HC, healthy control; HAMD-24, 24-item Hamilton Depression Scale; BDNF, brain-derived neurotrophic factor; VEGF, vascular endothelial growth factor.

The mediation analyses in MDD patients revealed that serum levels of BDNF and VEGF influenced the relative abundance of *Mogibacterium* in relation to HAMD-24 scores, after adjusting for age, sex, years of education, disease duration, and BMI (**Figure 3**). Specifically, both BDNF and VEGF exhibited a significant mediating effect on the association between the relative abundance of *Mogibacterium* and HAMD-24 scores (**Figure 3**). These findings suggest that peripheral neurotrophin may serve as mediating variables through which oral *Mogibacterium* abundance is linked to depression severity.

### Antidepressant treatment of MDD patients

Following the 2-week and 6-week antidepressant intervention, MDD patients showed significantly lower scores on the HAMD-24, SDS, and PSQI compared with baseline (**Table 1**). Furthermore, after adjusting for age, sex, years of education, disease duration, and BMI, a higher baseline relative abundance of Mogibacterium was significantly correlated with a smaller reduction in both HAMD-24 and SDS scores following 2-week and 6-week antidepressant treatment, as assessed by the rate of change from baseline (**Table 2**).

## Discussion

The main findings of the present study were as follows: (1) Significant differences in the diversity of the oral microbiota obtained from the buccal mucosa were observed between the MDD and HC groups. (2) Compared with HCs, MDD patients exhibited significantly reduced relative abundances of oral *Aggregatibacter*, *Lautropia*, and *Peptostreptococcus*, while the relative abundance of *Mogibacterium* was significantly increased. (3) Among these four genera, *Mogibacterium* demonstrated good performance in distinguishing MDD patients from HCs. (4) The relative abundance of oral *Mogibacterium* was significantly correlated with the severity of depressive symptoms and with serum levels of BDNF, NGF, and VEGF. (5) Serum BDNF and VEGF were found to influence the association between the relative abundance of *Mogibacterium* and MDD severity. (6) Following antidepressant treatment, the baseline relative abundance of oral *Mogibacterium* was significantly correlated with treatment efficacy at each time point, as reflected by changes in the HAMD-24 scores from pre- to post-treatment. These findings suggest that oral *Mogibacterium* from the buccal mucosa may serve as a potential biomarker for identifying MDD and predicting responses to antidepressant treatment. Furthermore, neurotrophin-related neuroplasticity may be involved in the association between oral *Mogibacterium* and MDD.

Although Zaura et al. (2014) indicated that the buccal mucosa and hard palate are two distinct niches (Zaura et al., 2014), a recent review classified these two sites into one microbial composition, as their microbiota were found to be similar using high-throughput DNA sequencing (Mark Welch et al., 2020). Since the bilateral buccal mucosa and hard palate are adjacent oral surfaces, this anatomical proximity allows for the frequent flow of microbes and promotes specific taxon-taxon interactions, supporting the notion that local habitat—including nearby taxa—is a key determinant of microbial niche (Mark Welch et al., 2020). In the present study, we therefore collected oral biospecimens from the bilateral buccal mucosa and hard palate using a single sterile swab. Combining these two oral sites for microbial analysis represents a methodological innovation of this study, reflecting an adaptation to the most recent research findings.

*Mogibacterium* is a genus of anaerobic bacteria commonly found in the oral microbial flora, notably as a component of dental plaque (Shikarkhane et al., 2024). It has been statistically significantly associated with periodontitis, as confirmed by meta-analysis (Antezack et al., 2023). Beyond its established role in periodontitis, oral *Mogibacterium* dysbiosis has been linked to an increased risk of various systemic conditions, including chronic obstructive pulmonary disease and gastrointestinal cancer (Jin et al., 2025; Liu et al., 2023). Furthermore, it is considered a core component of oral microbial dysbiosis in patients with Alzheimer’s disease (Weber et al., 2026), which, to date, represents the only evidence connecting oral *Mogibacterium* to CNS disorders. As a shared bacterium between the oral cavity and the gut, oral-to-gut translocation is proposed as the primary route for *Mogibacterium* dissemination in healthy individuals across all ages (Costa et al., 2024). This potential for translocation may explain its relevance in psychiatric and neurological conditions, as its presence in the gut has been implicated in other CNS-related disorders. For instance, the relative abundance of gut *Mogibacterium* was found to be significantly higher in patients with schizophrenia compared to healthy controls (Li et al., 2020), and its role has also been suggested in the pathophysiology of Parkinson’s disease (Ilie et al., 2025). The present study found that patients with MDD had a significantly higher relative abundance of oral *Mogibacterium* than healthy controls, a measure that showed acceptable diagnostic power. Moreover, this bacterial abundance was significantly correlated with the severity of depressive symptoms, as measured by HAMD-24, SDS, and PSQI scores, and specifically with circadian rhythm disturbances, somnipathy, and hopelessness. Oral *Mogibacterium* from the buccal mucosa may therefore show promise as a biomarker for both diagnosing MDD and objectively assessing symptom severity.

In the current study, a significant association was identified between the relative abundance of *Mogibacterium* and serum levels of GFAP. However, the mediation analysis did not yield statistically significant results regarding their relationship. Consequently, although we hypothesize that *Mogibacterium* may also play a role in neuroinflammation, it is possible that it is linked to other pathological processes underlying MDD. Emerging evidence implicates the gut microbiota as a critical regulator of neuroplasticity through the gut-brain axis, underscoring its significance in psychiatric and neurological health (Damiani et al., 2023; Mu and Wang, 2025). Accumulating evidence from animal and human research suggests that targeting the microbiota through probiotics or dietary changes could enhance neuroplasticity and offer therapeutic benefits for mental health (Al Noman et al., 2025). Impaired neuroplasticity is recognized as a core pathological feature of MDD; however, whether and how the oral microbiota contributes to this process remains unclear. To investigate this, we measured serum levels of five neurotrophins previously identified by our meta-analysis as strongly associated with MDD biology and treatment response (Shi et al., 2020). We found that the relative abundance of oral *Mogibacterium* was significantly correlated with serum BDNF, NGF, and VEGF levels in MDD patients, suggesting a potential link between oral microbial dysbiosis and neurotrophin-related neuroplasticity alterations. Moreover, the correlations between *Mogibacterium* and neurotrophins were significantly affected by depressive severity, suggesting that the associations between oral *Mogibacterium* dysbiosis and neurotrophins occur only under the condition of MDD. Large sample sizes and diverse groups of people are necessary to further determine whether MDD directly drives *Mogibacterium* dysbiosis-related neuroplasticity alterations, or whether oral *Mogibacterium* intrinsically regulates neuroplasticity under normal physiological conditions. Furthermore, the current mediation analysis revealed that peripheral BDNF and VEGF partially affected the association between oral *Mogibacterium* abundance and depression severity, implicating neuroplasticity as a possible mechanism by which oral microbiota may influence MDD pathophysiology (Wu et al., 2025). Notwithstanding previous findings that implicate the oral microbiota in MDD pathophysiology via BDNF DNA methylation (Girella et al., 2025), the potential role of oral microbiota, specifically *Mogibacterium*, in modulating BDNF and VEGF has yet to be elucidated. The current study offers evidence that the association between oral *Mogibacterium* and BDNF/VEGF in MDD patients is associational rather than causal. In addition, our previous study, based on the same cohort, reported that upregulation of oral Haemophilus expression may influence depressive symptoms, potentially through neuroinflammation. In the present study, additional correlation analyses revealed a significant association between the relative abundance of oral *Haemophilus* and serum BDNF levels in patients with MDD (r = -0.372, p = 0.021). Given that oral Since Given that *Haemophilus* dysbiosis originated from the same MDD patients reported in our previous study (Shi et al., 2025), this observed neurotrophin relationship is therefore not specific to *Mogibacterium* and likely reflects a generalized dysbiosis driven by depression severity as a shared upstream factor. As research on the oral microbiota in MDD remains in its early stages, the mechanisms through which the oral microbiota contributes to the pathogenesis of MDD may not be attributable to a single pathway. Therefore, while these findings contribute novel insights, their preliminary nature necessitates validation through future animal and molecular investigations.

To assess the influence of oral *Mogibacterium* on the antidepressant treatment, we further collected the data of neuropsychological assessments after clinical antidepressant treatment. At both the 2-week and 6-week follow-up points, the percentage reduction in HAMD-24 and SDS scores was significantly correlated with baseline oral *Mogibacterium* abundance. These findings suggest that pre-treatment oral *Mogibacterium* levels may serve as a predictor of symptomatic improvement following up to six weeks of antidepressant therapy in patients with MDD. While the current findings are based on a 6-week follow-up, future studies with extended observation periods are needed to evaluate the long-term predictive potential of pre-treatment oral *Mogibacterium* levels for antidepressant outcomes.

In this study, the relative abundances of oral *Aggregatibacter*, *Lautropia*, and *Peptostreptococcus* were significantly reduced in patients with MDD compared to HCs. A previous study reported increased relative abundances of oral *Aggregatibacter* and *Lautropia* in oral lichen planus, suggesting that these bacteria may be involved in oral chronic inflammation (Yan et al., 2024). *Peptostreptococcus*, a common anaerobic organism, exhibits increased abundance in chronic rhinosinusitis and other chronic inflammatory diseases (Brook, 2016; La Rosa et al., 2020). However, no current evidence supports a protective role for these oral bacteria against MDD. Additionally, no correlation was observed between the abundances of these three genera and *Mogibacterium* in MDD patients or HCs, suggesting that they may be not associated with the colonization of *Mogibacterium*. Furthermore, the higher abundances of these oral bacteria in HCs do not necessarily indicate that they are beneficial, as we did not compare their levels between HCs and individuals with chronic inflammatory diseases. Additionally, the current analysis shows that these bacteria have large standard deviations, suggesting substantial dispersion in the detection data, which may stem from the influence of sample quality, detection methods, and various environmental factors. Therefore, these findings should be interpreted with caution, particularly because it remains unclear whether the immune system of MDD patients affects the abundance of oral *Aggregatibacter*, *Lautropia*, and *Peptostreptococcus* under disease conditions. In the subsequent animal study, we will evaluate whether transplantation of these oral microbiota in animal models of depression could ameliorate the levels of *Mogibacterium* and depression-like behaviors.

There were some limitations in this study. (1) The antidepressant medications received by MDD patients were not consistent in this study, as the design was purely observational. Because baseline oral microbiome composition and treatment response may be affected by the specific class of antidepressant medication, we further compared the four subgroups based on medications. As shown in **Supplementary Figures 5**, **6**, no significant differences were observed among the four subgroups in either the baseline relative abundance of oral *Mogibacterium* or the rate of change in HAMD-24 scores at 2 and 6 weeks. However, given the limited sample size per group, which precluded robust statistical comparison, these findings should be interpreted with caution and validated in larger cohorts. In a follow-up study, we aim to conduct a rigorously controlled clinical intervention with a uniform medication regimen. (2) The present study was limited to a 6-week follow-up, representing the longest medication period assessed. The predictive value of oral *Mogibacterium* for longer-term antidepressant outcomes has yet to be determined, warranting future studies with extended observation. Furthermore, as oral samples were not collected post-treatment in this study, we were unable to analyze changes in oral *Mogibacterium* abundance after antidepressive treatment. To address this, future studies will employ multistage sampling and utilize metagenomic sequencing for the quantitative detection and monitoring of oral Mogibacterium levels in patients with MDD. (3) Both animal experiments and molecular studies are necessary to validate the causal role of oral *Mogibacterium* in depressive-like behaviors and to elucidate the neuroplasticity-related mechanisms underlying its association with MDD. In addition, as it is difficult to require uniform dietary habits among human subjects, we will adopt a standardized diet in animal experiments to minimize the influence of different dietary patterns on oral microbiota composition. (4) Due to this study just detected the serum levels of several neurotrophic factors, we cannot conclude that *Mogibacterium* contributes to the pathogenesis of MDD exclusively through mechanisms of neural plasticity. Neuroinflammation remains a plausible alternative pathway through which this microorganism may exert its effects. As a preliminary investigation, this study offers valuable insights into the potential role of oral microbiota in MDD. Further research is warranted to elucidate the underlying mechanisms of oral microbiota, which are likely to involve multiple pathways influencing brain function. (5) Based on strict inclusion and exclusion criteria and the study design, this study utilized a relatively small sample size. As a result, the current findings require further validation with larger samples. Meanwhile, gender may also impact the oral microbiota (Yamazaki et al., 2023); however, due to the relatively small overall sample size of this study, further stratified analysis by gender was not feasible. Consequently, we will conduct multicenter studies involving large, diverse populations to thoroughly assess the generalizability of the current research.

## Conclusion

This study identifies oral *Mogibacterium* dysbiosis from the buccal mucosa as a distinctive feature of MDD, characterized by significantly increased abundance in patients compared with healthy controls. This alteration not only distinguishes MDD patients with acceptable diagnostic accuracy but also correlates with depressive symptom severity, supporting its potential utility as a clinical biomarker. Furthermore, peripheral neurotrophins involved in neuroplasticity may represent a mechanistic link between oral *Mogibacterium* and MDD pathophysiology, although this finding remains exploratory and hypothesis-generating. Notably, pre-treatment *Mogibacterium* abundance predicts symptomatic improvement over six weeks of antidepressant treatment, highlighting its potential as a prognostic indicator of therapeutic outcomes.

## Data Availability

The datasets presented in this study can be found in online repositories. The names of the repository/repositories and accession number(s) can be found below: https://www.ncbi.nlm.nih.gov/, PRJNA1279610.
