# Supplementary material for: Potential value of oral Mogibacterium in major depressive disorder
Source: Front Cell Infect Microbiol. 2026 Apr 24;16:1816685. doi: 10.3389/fcimb.2026.1816685 (PMC13153035; doi:10.3389/fcimb.2026.1816685)
Supplement: Supplementary file 1 [file Table1.docx]

**Supplementary Methods**

**DNA extraction and PCR amplification**

Total microbial genomic DNA was extracted using the Bacterial DNA Extraction Mini Kit (Mabio, Guangzhou, China) according to manufacturer’s instructions. The quality and concentration of DNA were determined by 1.0% agarose gel electrophoresis and a NanoDrop® ND-2000 spectrophotometer (Thermo Scientific Inc., USA) and kept at -80 ℃ prior to further use. The hypervariable region V3-V4 of the bacterial 16S rRNA gene were amplified with primer pairs 338F (5'-ACTCCTACGGGAGGCAGCAG-3') and 806R (5'-GGACTACHVGGGTWTCTAAT-3') by an T100 Thermal Cycler (BIO-RAD, USA). All samples were amplified in triplicate. The PCR product was extracted from 2% agarose gel and purified. Then quantified using Synergy HTX (Biotek, USA). Using the NEXTFLEX Rapid DNA-Seq Kit (Bioo Scientific, USA) to generate the sequencing libraries. Purified amplicons were pooled in equimolar amounts and paired-end sequenced on an Illumina NextSeq 2000 PE300 platform (Illumina, San Diego,USA) according to the standard protocols by Majorbio Bio-Pharm Technology Co. Ltd. (Shanghai, China).

**Data processing**

Raw FASTQ files were de-multiplexed using an in-house perl script, and then quality-filtered by fastp version 0.19.6 and merged by FLASH version 1.2.11with the following criteria:

(i) the PE300 reads were truncated at any site receiving an average quality score of <20 over a 50 bp sliding window, and the truncated reads shorter than 50 bp were discarded, reads containing ambiguous characters were also discarded; (ii) only overlapping sequences longer than 10 bp were assembled according to their overlapped sequence. The maximum mismatch ratio of overlap region is 0.2. Reads that could not be assembled were discarded; (iii) Samples were distinguished according to the barcode and primers, and the sequence direction was adjusted, exact barcode matching, 2 nucleotide mismatch in primer matching. Then the optimized sequences were clustered into operational taxonomic units (OTUs) using Usearch 11 with 97% sequence similarity level. The most abundant sequence for each OTU was selected as a representative sequence. The number of 16S rRNA gene sequences from each sample were rarefied to 20,000, which still yielded an average Good’s coverage of 99.09% , respectively. The taxonomy of each OTU representative sequence was analyzed by RDP Classifier version 2.13 against the 16S rRNA gene database (e.g. Silva v138) using confidence threshold of 0.7. The metagenomic function was predicted by PICRUSt2 (Phylogenetic Investigation of Communities by Reconstruction of Unobserved States) based on OTU representative sequences.

Supplementary Table 1. ROC curve analyses

|  | AUC | 95% CI | Sensitivity | Specificity |
| --- | --- | --- | --- | --- |
| Aggregatibacter | 0.693 | 0.569 - 0.799 | 42.11% | 96.67% |
| Lautropia | 0.669 | 0.545 - 0.779 | 31.58% | 96.67% |
| Peptostreptococcus | 0.646 | 0.521 - 0.759 | 60.53% | 66.67% |
| Mogibacterium | 0.732 | 0.611 - 0.833 | 94.74% | 53.33% |

**Supplementary Table 2. Correlation analyses between relative abundance of** **Mogibacterium and serum neurotrophin levels in MDD patients, with controlling Age, sex, education years, body mass index, duration of the disease, and baseline HAMD-24 scores.**

|  | r-value | p-value |
| --- | --- | --- |
| Serum levels of BDNF | -0.217 | 0.196 |
| Serum levels of IGF-1 | 0.297 | 0.075 |
| Serum levels of NGF | -0.343 | 0.038 |
| Serum levels of VEGF | 0.186 | 0.271 |
| Serum levels of S100β | 0.108 | 0.524 |


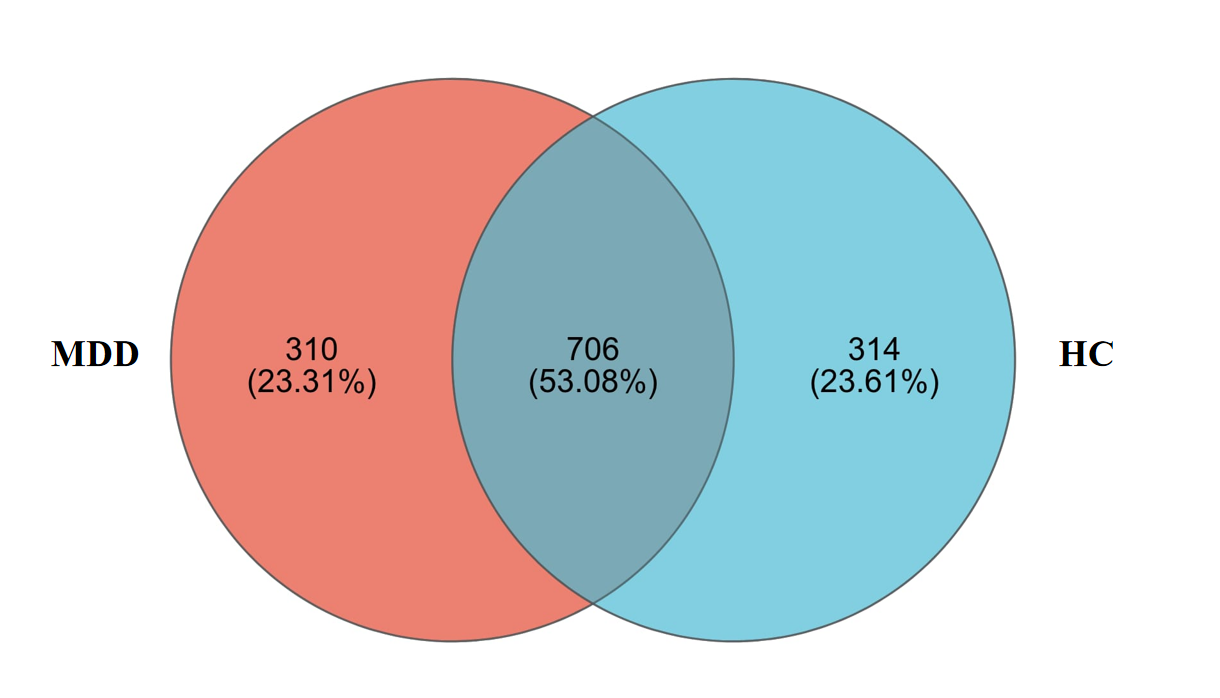


**Supplementary Figure 1. The number of OTUs in MDD and HC groups.**


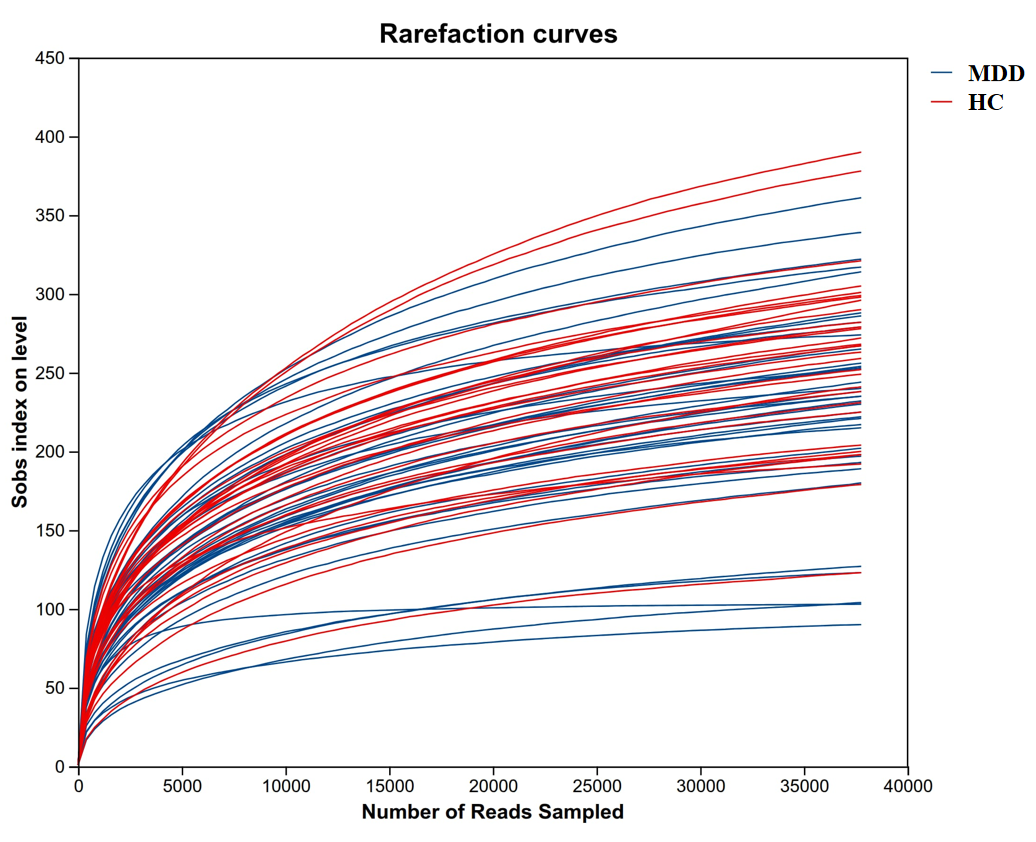


**Supplementary Figure 2. The rarefaction curves using α-diversity indices.**


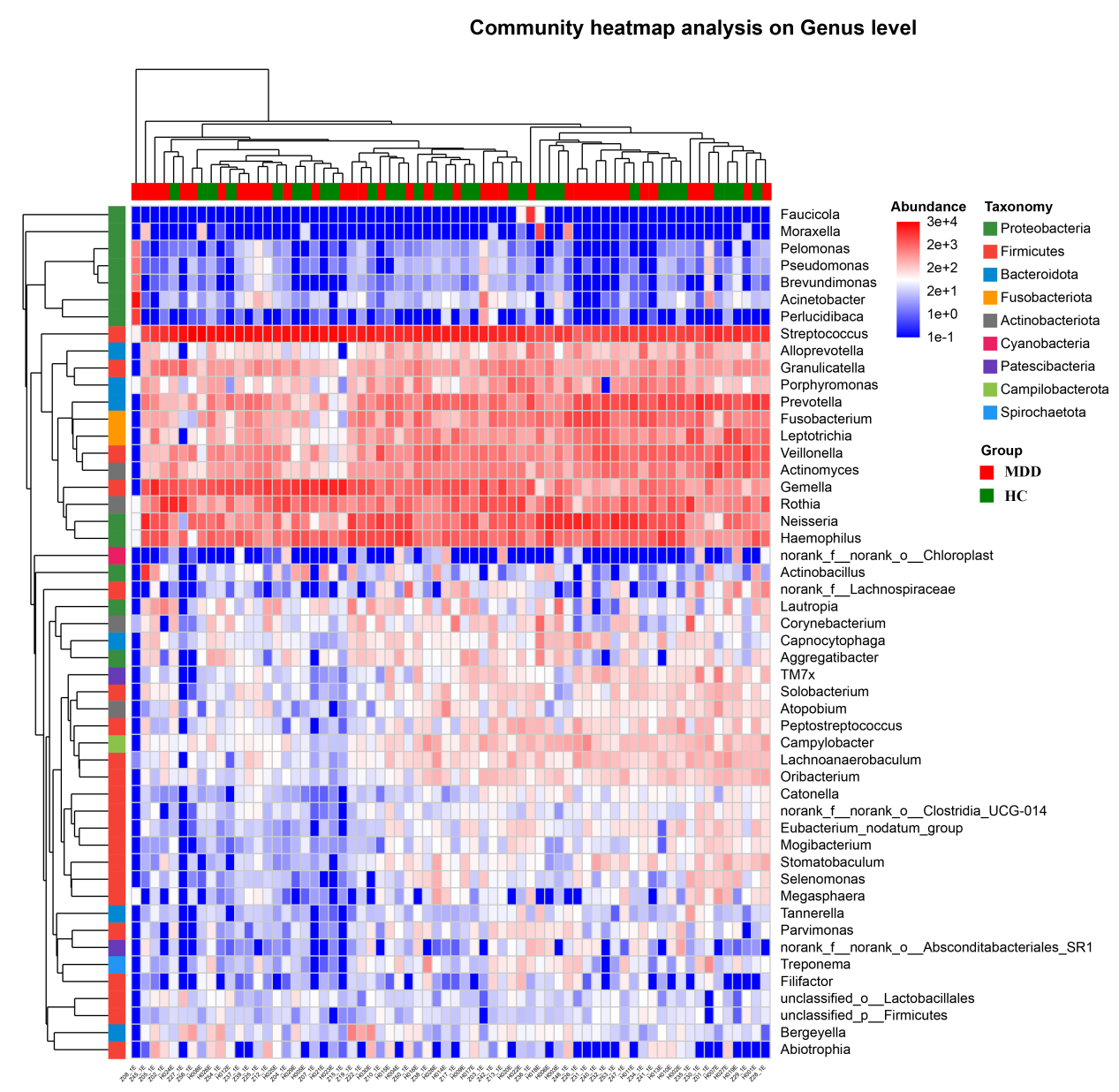


**Supplementary Figure 3. Heat-map analysis at genus (top 50).**

Abscissa is the sample and ordinate is the taxa at genus level. The colors in heat-map represent the species abundance.


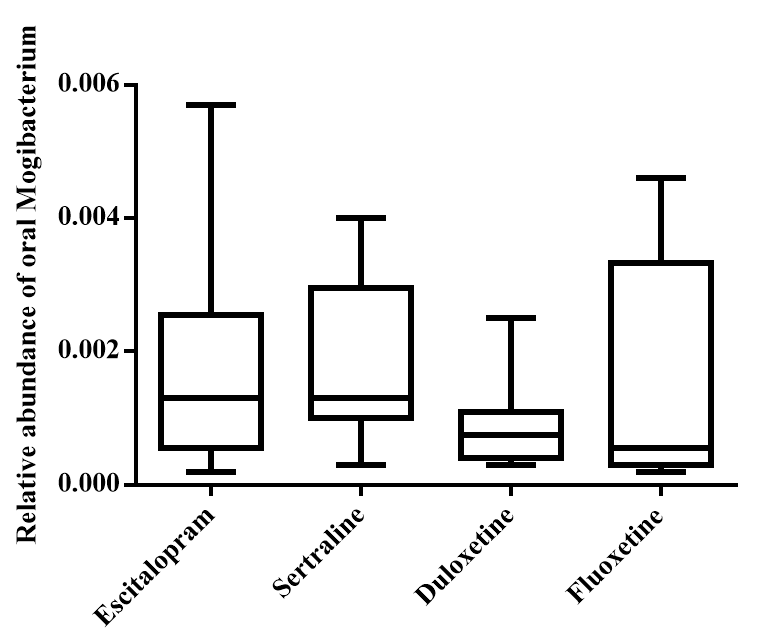


**Supplementary Figure 4. Difference of the baseline abundance of oral Mogibacterium among four kinds antidepressive drug subgroups** **in MDD patients**

Post-hoc analysis was performed using the Bonferroni correction.


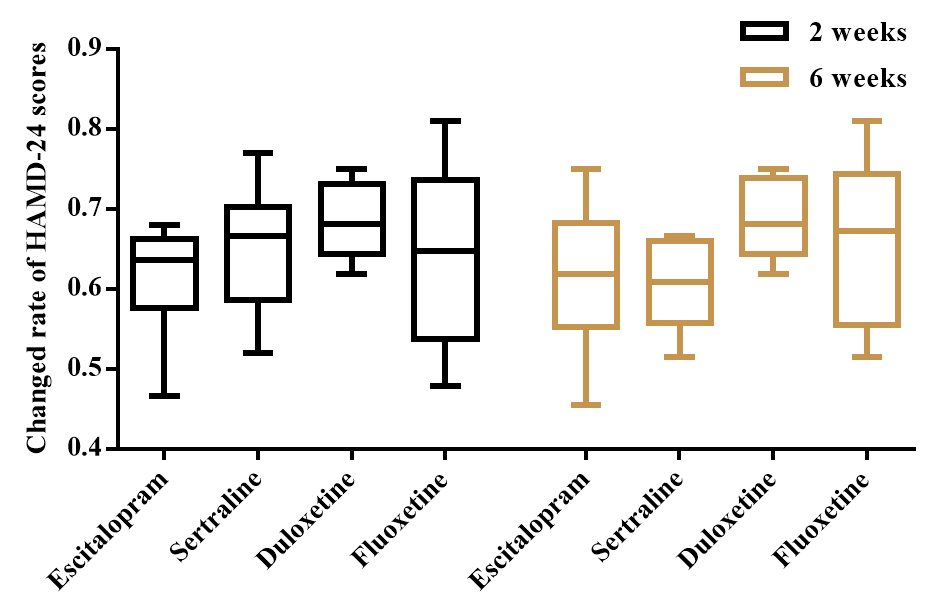


**Supplementary Figure 5. Difference of the changed rate of HAMD-24 among four kinds antidepressive drug subgroups** **in MDD patients during the follow-up.**

Post-hoc analysis was performed using the Bonferroni correction.
